# Subacute Thyroiditis after COVID-19: A Literature Review

**DOI:** 10.4269/ajtmh.21-1223

**Published:** 2022-09-06

**Authors:** Yuya Ando, Yosuke Ono, Azusa Sano, Naoya Fujita, Sachiko Ono

**Affiliations:** ^1^ Department of Internal Medicine, Division of Gastroenterology, Self-Defense Forces Central Hospital, Tokyo, Japan;; ^2^ Department of Family Medicine, Graduate School of Medical and Dental Sciences, Tokyo Medical and Dental University, Tokyo, Japan;; ^3^ Department of General Medicine, National Defense Medical College, Saitama, Japan;; ^4^ Department of Eat-loss Medicine, Graduate School of Medicine, The University of Tokyo, Tokyo, Japan

## Abstract

Subacute thyroiditis (SAT), potentially caused by severe acute respiratory syndrome coronavirus 2 infection, has been reported as a complication of COVID-19 since 2020. The clinical characteristics and outcomes of SAT after COVID-19 remain incompletely defined. Therefore, we aimed to collect and survey case reports of SAT after COVID-19. We performed a systematic search of PubMed/MEDLINE, Web of Science, and Google Scholar. The keywords and MeSH terms used for the searches were “subacute thyroiditis” and “COVID-19.” A total of 38 patients from 26 case reports, case series, and letters on SAT associated with COVID-19 were included and analyzed. The most frequent SAT symptom was neck pain (27 cases), followed by fever (22 cases). Of the 25 cases with information on the duration between onset of COVID-19 symptoms and onset of SAT symptoms, the shortest was simultaneous occurrence, and the longest was 4 months. In most cases, patients developed SAT at several days or weeks after the onset of COVID-19. All patients with SAT recovered with no severe complications or sequelae. Clinicians should be aware of the possibility of SAT development in patients with neck pain and fever following COVID-19. Further research is necessary to determine the relationship between SAT and COVID-19.

## INTRODUCTION

The COVID-19 pandemic, caused by SARS-CoV-2, has devastated the world since its emergence in 2019.^1^ Although the leading cause of death associated with COVID-19 is acute respiratory distress syndrome,^2^ other complications associated with COVID-19 have been reported, including acute kidney injury, pulmonary embolism, acute myocarditis, and septic shock.^3^

Currently, subacute thyroiditis (SAT), potentially invoked by SARS-CoV-2 infection, has been reported as a possible complication associated with COVID-19.^4^^,^^5^ SAT can be caused by multiple triggers, including viral infection, post-inflammatory processes, and autoimmunity.^6^^,^^7^ Patients with SAT exhibit fever, neck pain, and a tender diffuse goiter as well as abnormalities in serum thyroid hormones. The clinical characteristics and outcomes of SAT after COVID-19 remain incompletely defined.

In the present study, we gathered information on SAT after COVID-19 from case reports available in digital databases and described its clinical practice patterns, clinical symptoms, and outcomes. We aimed to examine the collected data on SAT after COVID-19 to further understand the relationship between SAT and COVID-19.

## METHODS

### Oversight and search strategy.

In accordance with the Preferred Reporting Items for Systematic Reviews and Meta-Analyses (PRISMA) Statement,^8^ we conducted a systematic search of PubMed/MEDLINE, Web of Science, and Google Scholar. References in the selected articles were reviewed manually and crosschecked for other relevant reports. The search of PubMed/MEDLINE was performed with the following MeSH terms: “COVID-19” and “Thyroiditis, Subacute.” The searches in Web of Science and Google Scholar were carried out with the following terms: “COVID-19” and “Subacute thyroiditis.” The full search terms were “COVID-19” [MeSH] AND “Thyroiditis, Subacute” [MeSH] and “COVID-19” AND “Subacute thyroiditis” in PubMed/MEDLINE; (covid) AND (subacute thyroiditis) in Web of Science; and (COVID-19) AND (subacute thyroiditis) AND (case report) in Google Scholar. The quality of the included articles was assessed using the CARE guidelines.^9^

### Selection of case reports.

The inclusion criteria for the case reports were as follows: 1) SARS-CoV-2 infection confirmed in the context, 2) diagnosis of SAT confirmed in the context, 3) article written in English, and 4) article published from 2020 to 2021. Cases were excluded if they had too little information, such as conference abstracts or autopsy reports only, or if they were duplicated. When results from a case were reported more than twice, the most recent case report was used. All case reports identified in the databases were screened for eligibility on title and abstract.

### Extraction of clinical information.

Y. A. conducted the extraction of clinical information from the literature with the assistance of Y. O. Data extracted for the cases included sex, age, past medical history of thyroid disease, race, onset date of COVID-19 symptoms, duration of COVID-19 symptoms, duration between onset of COVID-19 symptoms and onset of SAT symptoms, COVID-19 management, SAT management, steroid usage, information on recovery, and prognosis (died or survived).

## RESULTS

**Figure 1.**
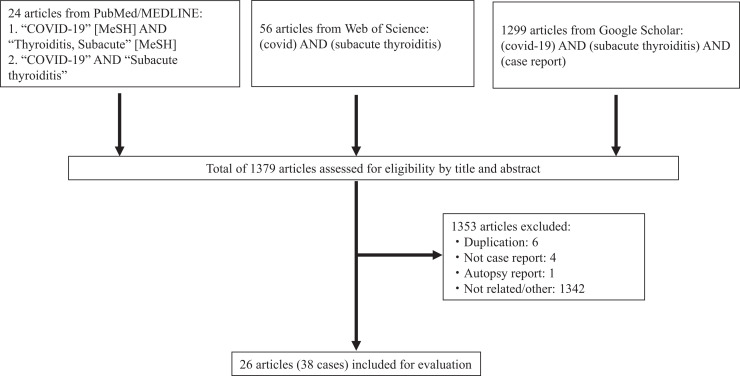
Flow chart of the case report selection process.

We identified 1,379 articles in our digital searches. After reviewing the titles and abstracts and excluding duplicates, we identified 38 cases in 26 articles published up to the end of December 2021.^4^^,^^5^^,^^10^^–^^33^ A flow chart of the article selection process is shown in Figure 1, and an overview of the 38 cases included in the study is provided in Table 1. The quality of the 15 case reports, four case series, six (scientific) letters, and one brief report was assessed and scored using the CARE guideline checklist (Supplemental Table 1).^9^ All 26 articles contained patient demographic information and diagnosis of SAT after COVID-19.

**Table 1:** Clinical characteristics of the patients with post–COVID-19 subacute thyroiditis

Ref no.	Age/ sex	Date of COVID-19 symptom onset or swab positivity	COVID-19 symptoms	Duration of COVID-19 symptoms	Severity	Treatment of COVID-19	Date of SAT onset after COVID-19	Duration between onset of COVID-19 symptoms and onset of SAT symptoms	SAT symptoms	Elevated inflammatory markers at SAT onset	Treatment of SAT	Steroid tapering or reduction	Symptom relief from SAT onset
4	38/F	March 1, 2020	Fever (37.5°C), rhinorrhea, anosmia, asthenia	4 days	Mild	n/a	March 17, 2020	16 days	Neck pain, asthenia, fever (38.5°C), palpitation, anorexia	ESR, CRP	Prednisone 25 mg/day	“At last evaluation (on May 10), while taking prednisone 15 mg/day, the patient was asymptomatic”	“Symptoms disappeared within a few days”
4	29/F	March 3, 2020	Rhinorrhea	3 days	Mild	n/a	April 2, 2020	30 days	Neck pain, asthenia, palpitation, sweating, fever (37.2°C)	ESR, CRP	Prednisone 25 mg/day Propranolol 40 mg/day	“At last evaluation (on May 18), while taking 15 mg/day of prednisone, the patient was asymptomatic”	“Neck pain and fever disappeared within 3 days and the other symptoms within 2 weeks”
4	29/F	March 17, 2020	Fever (38.0°C), cough, rhinorrhea, anosmia, diarrhea	14 days	Mild	n/a	April 22, 2020	36 days	Neck pain, palpitation, tachycardia, sweating,	n/a	Ibuprofen 600 mg/day	n/a	“Symptoms disappeared within 2 weeks”
4	46/F	April 3, 2020	Fever (38.2°C), cough, rhinorrhea, anosmia, asthenia	6 days	Mild	n/a	May 2, 2020	20 days	Neck pain, fever (37.2°c), palpitation, asthenia, insomnia, anxiety, weight loss	CRP	Prednisone 25 mg/day	“At the last evaluation (on June 15), while taking 20 mg/day of prednisone, the patient was asymptomatic”	“Neck pain and fever disappeared within a few days and other symptoms within 2 weeks”
5	69/F	n/a	Mild fever, cough, dyspnea	n/a	Moderate	Hydroxychloroquine, lopinavir, ritonavir, low-flow oxygen therapy	n/a	5 days after initiation of treatment of COVID-19	Palpitation, insomnia, agitation	n/a	Methylprednisolone 40 mg/day	“then continuing with 25 mg oral prednisone, to be progressively tapered over 4 weeks or more, according to clinical response”	“Within a few days, symptoms markedly improved”
10	18/F	February 28, 2020	Fever (37.5°C), rhinorrhea, cough, fatigue	4 days	Mild	No treatment	March 17, 2020	18 days	Fever (37.5°C), fatigue, neck pain, palpitation	ESR, CRP, WBC	Prednisone 25 mg/day	“Steroid was progressively tapered and at the last evaluation (April 27) while taking prednisone 16 mg/day, the patient was asymptomatic”	“Neck pain and fever disappeared within 2 days and the remaining symptoms within 1 week”
11	37/F	April 10, 2020	Odynophagia, anosmia	1 day	Mild	Symptomatic treatment	n/a	“A month after her initial presentation”	Neck pain, fatigue	ESR, CRP	n/a	n/a	“During her follow-up visit one month after diagnosis, the patient has remained asymptomatic”
12	49/M	n/a	Sore throat, fever, cough, shortness of breath	n/a	Moderate	Hydroxychloroquine 200 mg twice daily, enoxaparin 0.4 mL once daily	n/a	10 days post-discharge from infection control facility for COVID-19	Sore throat, swallowing difficulty, fever (38.3°c), “tonsils were hyperemic”	ESR, CRP, WBC	Methylprednisolone 32 mg/day	“steroid dose was gradually decreased and was planned to be completed in 4 weeks and called for control 1 month after.”	“At 1-week post-treatment, the patient was asymptomatic”
13	41/F	n/a	Fever (38.5°C), neck pain	n/a	Mild	Hydroxychloroquine tablet 200 mg twice daily for 5 days	n/a	n/a	Fever (38.5°C), neck pain	ESR, CRP, WBC	Prednisolone 16 mg/day	“She was discharged on prednisolone tapering dose for 4 weeks”	“Prednisolone 16 mg daily was given and she showed significant improvement of clinical condition”
14	41/F	n/a	Fever, cough, coryza	n/a	Mild	Oral azithromycin, supportive therapy	n/a	4 weeks	Neck pain, fever (39.5°c), odynophagia, chills, diaphoresis, weight loss (6 kg), fatigue, alopecia, heat intolerance, irritability, headaches, bilateral hand tremors, palpitation	ESR, CRP	Ibuprofen 600 mg (every 6 h), prednisone 40 mg/day	“She completed a 4-week corticosteroid taper”	“complete symptom resolution at her last outpatient follow-up visit 45 days from hospital discharge (2 days of hospitalization)”
15	34/M	n/a	Fever, dry cough, headache, anosmia, sore throat	n/a	Mild	Paracetamol, dequalinium lozenges	n/a	9 days	Neck pain, tachycardia	CRP, WBC	Prednisolone 20 mg/day, atenolol 25 mg/day	“A steroid-tapering regimen was planned for him to decrease the dosage of prednisone to the minimum required for symptomatic relief with periodic monitoring of thyroid function.”	“He was reviewed after 10 weeks in the outpatient clinic. He had completed his tapering course of steroids and was clinically well with no symptoms.”
16	28/F	April 15, 2020	Diarrhea, abdominal pain	n/a	Mild	n/a	May 14, 2020	29 days	Fever (38.5°C), neck pain, sore throat, palpitation, asthenia	ESR, CRP, WBC	Aspirin 500 mg (every 6 h), propranolol 40 mg (every 6 h)	n/a	“Symptoms improved in 24 h, with a total relief in 2 weeks”
17	43/F	Beginning of March 2020”	Fever, rhinorrhea, painful swallowing, cough, hoarseness, conjunctivitis	n/a	Mild	No specific treatment	Mid-April 2020	6 weeks	Fever (37.5°C), pain and tenderness in the anterior cervical region, fatigue, tremors, palpitation	n/a	Prednisone 25 mg/day	“25 mg/day as the starting dose, gradually tapered”	“4 weeks after starting steroid therapy, all thyroid functional tests normalized, as well as inflammatory indexes”
18	47/F	n/a	“She did not have fever or respiratory symptoms but had right lower lobe pneumonia on chest radiograph”	n/a	Mild	Oral hydroxychloroquine, intravenous ceftriaxone	n/a	n/a	Anterior neck pain	CRP	Mefenamic acid, celecoxib	n/a	“The patient reported full resolution of symptoms a month later.”
19	26/F	n/a	Dry cough	1 week	Mild	n/a	n/a	n/a	Fever, fatigue, palpitation, anterior neck pain	CRP, ESR, WBC	n/a	n/a	n/a
19	37/F	n/a	Myalgia	Few days	Mild	n/a	n/a	n/a	Fever, fatigue, palpitation, anterior neck pain	CRP, ESR, WBC	n/a	n/a	n/a
19	35/M	n/a	No symptoms	n/a	Mild	n/a	n/a	n/a	Fever, fatigue, palpitation, anterior neck pain	CRP, ESR, WBC	n/a	n/a	n/a
19	41/F	n/a	Low-grade fever, mild myalgia	Few days	Mild	n/a	n/a	n/a	Fever, fatigue, palpitation, anterior neck pain	CRP, ESR, WBC	n/a	n/a	n/a
19	52/M	n/a	Low-grade fever, dry cough, mild myalgia	Few days	Mild	n/a	n/a	n/a	Fever, fatigue, palpitation, anterior neck pain	CRP, ESR, WBC	n/a	n/a	n/a
19	34/F	n/a	No symptoms	n/a	Mild	n/a	n/a	n/a	Fever, fatigue, palpitation, anterior neck pain	CRP, ESR, WBC	n/a	n/a	n/a
20	64/M	n/a	Oppressive chest pain, fever, productive cough, headache, fatigue	14 days	Moderate	Steroids, azithromycin, Acetaminophen	n/a	“Three weeks after the first positive PCR test for COVID-19”	Distal tremor, diaphoresis	Ferritin	Atenolol 50 mg twice daily, prednisone 50 mg	“gradual dosage tapering over the course of 2 weeks”	“Three days after admission, the patient was discharged with full clinical relief. On his last outpatient visit to the endocrine clinic, he had discontinued steroids. His thyroid tests revealed a slight primary hypothyroidism, which was treated with 100 μg of levothyroxine.”
21	58/M	June 6, 2020	Fever, myalgia, fatigue	n/a	Moderate	Azithromycin, paracetamol	June 12, 2020	6 days	Fever, fatigue, delirium, staring gaze, weakness, weight loss, tender thyroid, odynophagia, tachycardia	ESR, CRP, WBC, ferritin	Dexamethasone (4 mg), naproxen oral prednisolone (15 mg)	“Stepwise tapering off”	8 days
22	37/M	n/a	Cough, chills, fever, dyspnea	7 days	Mild	Supportive care	n/a	1 month	Anterior neck pain, fatigue, chills, postural tremor, palmar erythema, tachycardia	ESR, CRP	Aspirin, propranolol	n/a	1 week hypothyroidism
23	58/M	n/a	n/a	n/a	Mild	Analgesics, favipiravir, azithromycin, zinc tablets, vitamin c capsules	n/a	n/a	Fever, tachycardia, neck pain, increased stool frequency	ESR, CRP	Prednisolone 30 mg/day, propranolol 40 mg/day	“gradually tapered over next 1 month and then stopped”	Hypothyroidism
24	44/M	n/a	Shortness of breath, loose stools, fatigue, hypoxia, fever, dyspnea	5 months	Severe	15 L of oxygen, intravenous antibiotics and fluids	n/a	n/a	Malaise, odynophagia, neck pain	ESR, CRP, WBC	Propranolol 40 mg, paracetamol, ibuprofen	n/a	6 weeks
25	45/F	n/a	Nasal congestion, cough, weakness, muscle pain, headache	7 days	Moderate	“Symptomatic and antibacterial therapy”	n/a	30 days	Neck pain, fever, myalgia, palpitation, sweating, tachycardia	ESR, WBC	Prednisone 30 mg/day	“Reduction in the dosage by 5 mg weekly”	Within a week
25	40/F	n/a	n/a	n/a	Moderate	n/a	n/a	30 days	Neck pain, fever, palpitation, tachycardia	CRP, ESR, WBC	Prednisone 30 mg/day	“Reduction in the dosage by 5 mg weekly”	Within a week
26	29/F	n/a	n/a	n/a	n/a	Azithromycin, hydroxychloroquine	n/a	7 weeks	Fever, odynophagia, tachycardia, shortness of breath, weight loss, anterior neck tenderness, hand tremor	ESR, CRP	Prednisone 20 mg/day, atenolol 25 mg/day; “her prednisone was increased to 40 mg and atenolol to 50 mg daily”	“The prednisone was gradually tapered off over six weeks”	“The patient remained asymptomatic at ten weeks follow up”
27	39/M	n/a	Sore throat, fatigue, fever	7 days	Mild	Favipiravir	December 28, 2020	n/a	Neck pain, fatigue, muscle pain, palpitation, tremors, sinus tachycardia	ESR, CRP, WBC	Prednisolone 16 mg/day, ibuprofen 1200 mg/day, propranolol 20 mg tid	“reduced consecutively”	1 week
28	31/F	n/a	Fever, painful sore throat	n/a	Mild	“No specific treatment”	n/a	2 weeks	Anterior neck pain, fever, malaise	CRP, ESR, WBC	NSAIDs, prednisolone 15 mg/day	7 weeks	1 day
29	41/F	September 15, 2020	Headache, fatigue, loss of appetite, fever	n/a	Mild	“Symptomatic treatment”	n/a	6 weeks	Palpitation, insomnia	n/a	n/a	n/a	“Three weeks later, she developed hypothyroidism”
30	67/M	n/a	Shortness of breath, diarrhea	n/a	Severe	Ceftriaxone, azithromycin	n/a	n/a	Arrhythmia, weight loss, fatigue, diarrhea	ESR, CRP	Prednisone 20 mg/day	9 months	n/a
31	33/M	September 23, 2020	Fever (39°C), sore throat, body aches, lethargy, chills, sweating, dry cough, tachycardia	n/a	Moderate	Acetaminophen, naproxen, diphenhydramine, remdesivir, enoxaparin	October 2, 2020	9 days	Sore throat, neck tenderness	ESR, CRP, IL-6	Dexamethasone 4 mg every 8 hours for 5 days, prednisone 25 mg/day	“He was discharged with oral prednisone 25 mg daily with taper prescribed”	7 weeks
32	50/M	October 6, 2020	Neck pain, fever (39°C), headaches, cough, malaise, loss of smell, retrosternal discomfort	n/a	n/a	Azithromycin Amoxicillin Dexamethasone	6 October 2020	Simultaneously	Fever, cough, headache, insomnia, neck pain, hard neck tumor	CRP, ESR	Prednisone	Gradual dose reduction	“No symptom recurrence was observed for 10 months of follow-up”
32	39/F	December 2020	Cough, headache, fever, malaise	n/a	n/a	n/a	n/a	4 weeks	Neck pain, tachycardia, insomnia, tremor, fever	CRP, ESR	Prednisone 60 mg/day	4 months	“No recurrence was observed for the 8 months of follow-up”
32	55/F	n/a	“typical clinical symptoms”	n/a	n/a	n/a	n/a	5 weeks	Neck pain, fever	CRP, ESR	Prednisone	n/a	n/a
32	57/F	October 2020	“The disease course was mild with common symptoms”	n/a	n/a	n/a	n/a	4 months	Neck pain, fever, tachycardia, mood change	CRP, ESR	Prednisone 40 mg/day	“Gradual dose reduction”	n/a
33	46/F	n/a	Asymptomatic	n/a	Mild	Untreated	n/a	n/a	Neck pain, earache, fever, malaise, insomnia	ESR	NSAIDs, prednisone 40 mg/day	Six weeks	Two weeks

CRP = C-reactive protein; ESR = erythrocyte sedimentation rate; F = female; IL-6, interleukin-6; M = male; n/a = not available; NSAID = nonsteroidal antiinflammtory drug; PCR = polymerase chain reaction; Ref = reference; SAT = subacute thyroiditis; WBC = white blood cell count.

Of the 38 patients with SAT after COVID-19, 25 were women. In the four case reports that described patient race, two patients were Caucasian and two were Asian. Among the total patients, two had past medical history of thyroid disease, described as nontoxic goiter. We confirmed SARS-CoV-2 infection in the context from a swab or laboratory examination in 20 cases. COVID-19 symptoms were confirmed for 32 cases. The most frequent COVID-19 symptom was fever (22 cases). Three cases were described as asymptomatic. Three case reports did not describe the COVID-19 symptoms. On the basis of the living guidance for clinical management of COVID-19 provided by the WHO,^34^ 23 cases were categorized as mild, seven cases as moderate, and two as severe.

All reports for the 38 cases described SAT symptoms. The most frequent SAT symptom was neck pain (27 cases), followed by fever (22 cases). Of the 25 cases with information on the duration between onset of COVID-19 symptoms and onset of SAT symptoms, as mentioned in the text or able to be calculated, the shortest was simultaneous occurrence (0 days) and the longest was 4 months. In most cases, patients developed SAT at several days or weeks after the onset of COVID-19.

We confirmed that the thyroid state was described for 22 cases. Enlarged thyroid was the most common state (11 cases), followed by any tenderness (nine cases). We further confirmed that 37 patients had thyrotoxicosis, evaluated by detection of free T4, free T3, or thyroid-stimulating hormone. One article did not mention the actual serum levels of thyroid hormones, but we concluded that the patient experienced SAT from the following text: “Thyroid function tests were consistent with subclinical hypothyroidism.”^4^ The states for thyroglobulin, thyroglobulin antibody, antithyroid peroxidase antibody, and thyroid-stimulating hormone receptor antibody were mentioned in 27 cases. We further extracted the results of biological examinations reported for 35 cases. Of these 35 cases, 31 cases had increased C-reactive protein, and 30 cases had confirmed elevation of erythrocyte sedimentation rate. Interleukin-6 was measured in only one case and was found to be elevated.^31^ Ultrasound analysis of the thyroid was carried out in 14 cases and revealed hypoechoic areas in an enlarged thyroid in most cases. These findings were consistent with SAT.

Management of SAT was reported for 30 cases. Twenty-five patients were treated with corticosteroids such as prednisone. All patients recovered from both COVID-19 and SAT, although four patients suffered from hypothyroidism after SAT.^20^^,^^22^^,^^23^^,^^29^

## DISCUSSION

In this study, we analyzed 38 cases of SAT after COVID-19. Most patients developed SAT at several days or weeks after the onset of COVID-19. However, some cases had onset of SAT at several months after the onset of COVID-19. In most SAT cases, the severity of COVID-19 was categorized as mild. The symptoms associated with SAT were similar to those previously reported for typical SAT cases.^35^^,^^36^ Although four patients suffered from hypothyroidism after SAT, none of the patients died of SAT.

We found that the clinical characteristics of SAT after COVID-19 were similar to those of typical SAT. In general, young female adults are commonly affected by SAT,^36^^,^^37^ and the cases in the present study were consistent with this trend. The present study also demonstrated that the biological data for SAT after COVID-19 resembled those for typical SAT. These similarities suggest that SAT associated with COVID-19 may occur with the same pathophysiology as typical SAT, including viral infection, post-inflammatory processes, and autoimmunity. To date, the incidence rate of SAT after COVID-19 remains unknown. Further epidemiological research should be undertaken to determine the relationship between SAT and COVID-19.

Our study showed that SAT can develop regardless of the severity of COVID-19. Previous reports indicated the importance of IL-6 in the cytokine storm associated with COVID-19.^38^^,^^39^ Direct damage to the thyroid gland was also hypothesized.^40^ However, in most SAT patients, the severity of COVID-19 was categorized as mild or moderate.

Most patients with SAT were treated with steroids, followed by gradual tapering over several months. It is noteworthy that all the patients in our study became asymptomatic within several months. Previous reports described that some patients with COVID-19 had sequelae, such as fatigue, dyspnea, chest pain, and cough.^41^^,^^42^ Patients with COVID-19 can also have psychological or cognitive symptoms.^43^^,^^44^ In our study, all patients with SAT following COVID-19 had no remarkable sequelae after their recovery.

### Thyroid and COVID-19.

Currently, several reports have suggested an underlying relationship between the thyroid and COVID-19. Expression of angiotensin-converting enzyme 2 receptor was detected in the thyroid,^45^^,^^46^ leading to a hypothesis that SARS-CoV-2 directly infects the thyroid tissue.^47^ This may arise because angiotensin-converting enzyme 2 receptor and cellular protease TMPRSS2 are the SARS-CoV-2 entry requirements for infection.^48^^,^^49^ Some researchers mentioned the anatomical location of the thyroid, being near the front of the airway and lying against and around the front of the larynx and trachea.^47^^,^^50^ Specifically, this anatomical location can provide an easy entry point for the virus.

### Strengths and limitations.

The strength of our study is the relatively large number of cases compared with previous studies because we accumulated cases until the end of December 2021. In addition, the WHO staging was applied.^34^ Our study showed that SAT can occur regardless of the severity of COVID-19, thus alerting physicians that SAT can develop under COVID-19 therapy.

The present study has some limitations. First, the number of cases collected was too small to reach a conclusion on the relationship between SAT and COVID-19. Second, the descriptive quality of the case reports was heterogeneous, which made it difficult to review the reports comprehensively. Because the effect of publication bias was strong and statistical analysis will result in low generalizability, we refrained from conducting statistical analyses. Third, publication bias could be inevitable in this research because there are likely unpublished cases of SAT after COVID-19. This may arise because SAT is not always critical and may be overlooked by physicians. Finally, bias arising from the database or language choices may have affected the case selection.

Nevertheless, given the increasing numbers of COVID-19 cases worldwide, it is estimated that the number of cases with SAT after COVID-19 infection will increase. The present research enables physicians to consider certain clinical implications, such as when to suspect SAT in COVID-19 patients or when to prescribe agents. In addition, we suggest the importance of monitoring serum levels of thyroid hormones in COVID-19 patients.

### Conclusion.

In conclusion, SAT may develop days or weeks after the onset of COVID-19. The clinical features of SAT after COVID-19 were similar to those of typical SAT, and in most cases, the patients achieved complete remission using steroids without sequelae. Clinicians should be aware of the possibility of SAT in patients with neck pain and fever after COVID-19.

## Supplemental files


Supplemental materials
