# Supplementary material for: Subacute Thyroiditis after COVID-19: A Literature Review
Source: Am J Trop Med Hyg. 2022 Sep 6;107(5):1074–82. doi: 10.4269/ajtmh.21-1223 (PMC9709026; doi:10.4269/ajtmh.21-1223)
Supplement: Supplementary file 1 [file tpmd211223.SD1.pdf]

Supplementary Table 1: Scores for the case reports according to the CARE guideline checklist

Ref no.	Title	Key words	Abstract *	Introduction	Patient information*	Clinical findings	Timeline	Diagnostic assessment*	Therapeutic intervention**	Follow-up and outcomes*	Discussion *	Patient perspective	Informed consent	Article type	Total score
4	0	1	2	0	2	1	1	2	2	2	2	0	0	CS	15
4	0	1	2	0	2	1	1	2	2	2	2	0	0	CS	15
4	0	1	2	0	2	1	1	2	2	2	2	0	0	CS	15
4	0	1	2	0	2	1	1	2	2	2	2	0	0	CS	15
5	1	1	0	0	3	1	0	3	3	2	2	0	1	Letter	17
10	0	1	4	1	2	1	1	2	2	1	3	0	0	CR	18
11	0	0	2	0	3	1	0	2	0	2	2	0	0	CR	14
12	1	1	3	0	3	1	0	2	2	2	3	0	1	CR	19
13	0	0	0	0	3	1	0	3	2	1	3	0	1	Letter	14
14	1	1	4	1	4	1	0	2	3	2	2	0	1	CR	22
15	0	0	4	1	3	1	0	3	2	2	1	0	1	CR	18
16	0	0	0	0	3	1	0	2	2	2	3	0	0	Letter	13
17	0	1	3	0	3	1	0	2	2	2	3	0	1	Letter	18
18	0	0	4	1	3	1	0	2	2	2	3	0	1	CR	19
19	0	1	4	0	2	1	0	2	0	1	3	0	1	BR	15
19	0	1	4	0	2	1	0	2	0	1	3	0	1	BR	15
19	0	1	4	0	2	1	0	2	0	1	3	0	1	BR	15
19	0	1	4	0	2	1	0	2	0	1	3	0	1	BR	15
19	0	1	4	0	2	1	0	2	0	1	3	0	1	BR	15
19	0	1	4	0	2	1	0	2	0	1	3	0	1	BR	15
20	0	1	3	1	2	1	0	2	2	3	3	0	0	CR	18
21	0	1	2	1	4	1	1	2	2	3	4	0	0	CR	21
22	0	0	0	1	3	1	0	2	3	2	3	0	0	Letter	15
23	1	0	3	0	4	1	0	3	2	3	4	1	1	CR	23
24	1	1	2	1	4	1	0	2	2	2	3	0	1	CR	20
25	0	1	2	1	3	1	0	2	2	2	3	0	1	CS	18
25	0	1	2	1	2	1	0	2	2	2	3	0	1	CS	17
26	0	1	3	1	4	1	0	2	3	2	2	1	1	CR	21
27	1	1	2	0	3	1	0	3	2	3	3	0	0	CR	19
28	0	1	3	1	3	1	1	2	2	2	3	0	0	CR	19
29	0	0	4	0	2	0	0	2	2	2	3	0	1	CS	16
30	0	1	4	1	3	1	1	2	2	2	3	0	1	CR	21
31	1	1	3	1	2	1	0	2	2	2	3	0	1	CR	19
32	0	1	3	1	2	1	0	2	2	2	3	0	1	CS	18
32	0	1	3	1	2	1	0	2	2	2	3	0	1	CS	18
32	0	1	3	1	2	1	0	2	2	2	3	0	1	CS	18
32	0	1	3	1	2	1	0	2	2	2	3	0	1	CS	18

33	1	0	0	1	3	1	1	1	3	3	3	0	0	Letter	17

Abbreviations: Ref no., reference number; n/a, not available; BR, brief report; CR, case report; CS, case series.

A score of 0 means “none”.

A score of 1 to 4 means the number of matches to each checklist.
